# Carboplatin-Induced Hematuria With Obstructive Acute Kidney Injury

**DOI:** 10.7759/cureus.58931

**Published:** 2024-04-24

**Authors:** Naveenkumar Nallathambi, Adithyan Chinnadurai, Yogesh S.

**Affiliations:** 1 Internal Medicine, Madras Medical College, Chennai, IND; 2 Internal Medicine, Rajiv Gandhi Government General Hospital, Chennai, IND

**Keywords:** chemotherapy-related toxicity, urinary obstruction, acute kidney injury, hematuria, carboplatin

## Abstract

Platinum-based chemotherapeutic agents such as cisplatin, carboplatin, and oxaliplatin are used as adjuvant or neoadjuvant agents in malignancies of the ovary, cervix, lymphoma, head and neck, and breast. Cisplatin is most commonly used until the carboplatin is approved by the Food and Drug Administration (FDA). Cisplatin is not tolerated in many patients due to severe nausea and renal tubular injury. Carboplatin is used in patients where side effects limit the uses of cisplatin. Although carboplatin is least commonly associated with hematuria, we report a case of carboplatin-induced hematuria with obstructive acute kidney injury (AKI). Our patient, a 63-year-old female diagnosed with triple-negative breast carcinoma and post-mastectomy, was started on adjuvant chemotherapy, with carboplatin 700 mg and paclitaxel 250 mg. She developed hematuria with ureter obstruction due to clots, resulting in obstructive AKI. The patient continued to have oliguria and worsening symptoms, and thus, the ureter was stented. The patient's renal function returned to the baseline. In this case, we highlight the fact that carboplatin can cause hematuria with ureter obstruction. Adequate hydration before infusing carboplatin as in cisplatin can reduce the complications.

## Introduction

Platinum-based compounds are widely used as neoadjuvant or adjuvant chemotherapy in various malignancies of the ovary, cervix, lymphoma, head and neck, and breast carcinoma. The mechanism of action involves the formation of crosslinks, peroxidation, and cell apoptosis [1]. The compounds in this group are cisplatin, carboplatin, and oxaliplatin. Cisplatin is associated with severe nausea and nephrotoxic. Carboplatin is used in patients who are not tolerant to cisplatin. However, it can lead to several adverse effects, including nephrotoxicity [2]. One rare but potentially severe complication of carboplatin therapy is hematuria with obstructive acute kidney injury (AKI), which can significantly impact patient outcomes and quality of life.

Carboplatin-induced hematuria with obstructive AKI is thought to occur due to the formation of obstructive clots within the renal collecting system, leading to impaired urine flow and subsequent kidney injury [3]. The exact pathophysiology of this condition is not fully understood, but it is believed to involve the direct toxic effects of carboplatin on the renal tubules and the activation of coagulation pathways [4].

Management of obstructive AKI includes supportive measures to maintain renal function and alleviate obstructive symptoms. In severe cases, interventions such as ureteral stenting or percutaneous nephrostomy may be necessary to relieve urinary obstruction. Here, we discuss a similar case report, underscore the importance of preparedness for this adverse event with carboplatin, and highlight adequate hydration could help in prevention.

## Case presentation

**Figure 1:**
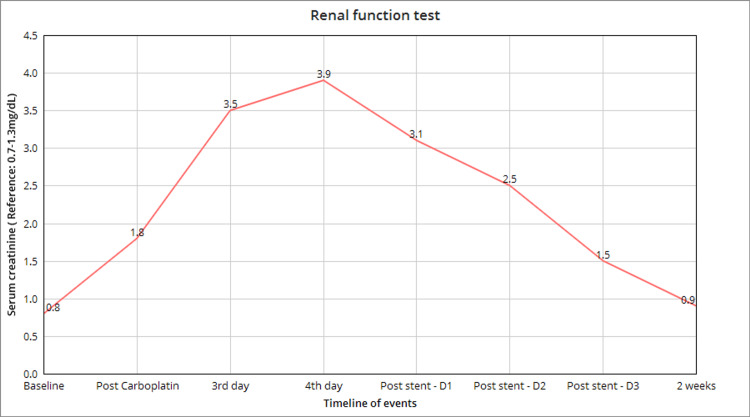
Trend graph showing the rise of creatinine after carboplatin and return to baseline after ureter stenting.

Our patient was a 63-year-old female with a past medical history of type 2 diabetes and hypertension with no significant family history. She had a history of alcohol and nicotine use in the past. She had a lump in her breast for six months. On clinical examination, a 6 cm x 10 cm mass was palpable in the left breast with palpable axillary lymph nodes. She underwent an excision biopsy of the lump with lymph node dissection, and the biopsy revealed triple-negative breast malignancy (ER-, PR-, and HER2-). Owing to the lymph nodal involvement and basal type of breast carcinoma, adjuvant chemotherapy with 700 mg carboplatin and 250 mg paclitaxel was initiated. The patient developed gross hematuria after receiving the carboplatin on the following day. Then the patient developed loin pain progressed to a decrease in urine output. Baseline investigations revealed anemia, and creatinine was normal before initiation of carboplatin (Figure 1; Table 1). After receiving carboplatin, renal functions started to decline with increasing creatinine.

**Table 1:** Laboratory analysis of hemogram and the renal function test.

Parameter	Observed value	Reference values
Hemoglobin	11.2 g/dL	11.5-15.5 g/dL
White cell count	6,000 mm^-3^	5,000-10,000 mm^-3^
Platelet	250 x 10^9^/L	150 x 10^9^ to 400 x 10^9^/L
Sodium	141 mEq/L	136-145 mE/L
Potassium	3.8 mmol/L	3.5-5 mmol/L
pH	7.38	7.35-7.45
HCO3-	22 mEq/L	22-26 mEq/L
PCO2	38 mmHg	35-45 mmHg
Uric acid	5 mg/dL	3.5-7.2 mg/dL
Phosphorus	3.2 mg/dL	2.8-4.5 mg/dL

**Figure 2:**
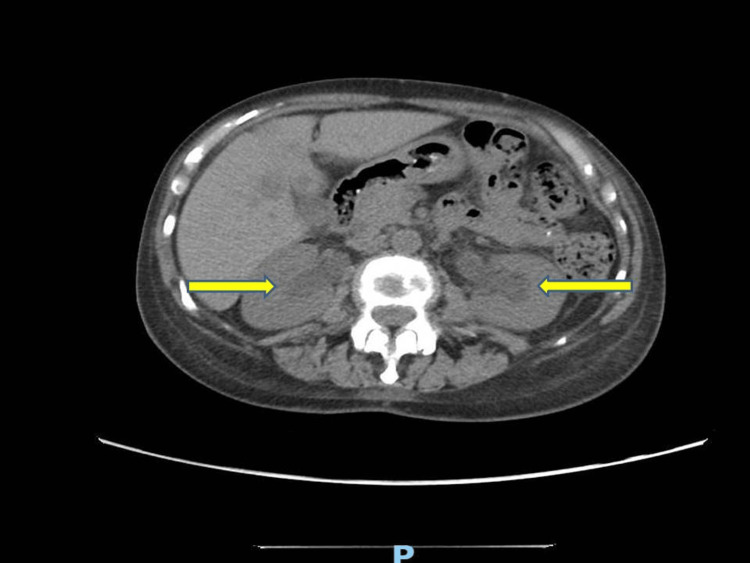
CT abdomen: yellow arrows demonstrating the bilateral hydroureteronephrosis.

An ultrasonogram of the abdomen demonstrated hydroureteronephrosis and an empty bladder. CT scan of the abdomen showed bilateral hydroureteronephrosis, with an isodense obstruction within the upper ureter, the bladder was empty, suggesting the possibility of ureteral obstruction, as seen in Figure 2.

Urine output remained around 300-400 mL/day. Despite declining renal function, she had urine output with no evidence of hyperkalemia and metabolic acidosis. Hence, the renal replacement therapy was not initiated. Given ureteral obstruction due blood clot, she was initially managed symptomatically with intravenous analgesics. The patient continued to exhibit a rise in creatinine levels and worsening loin pain. Ureteral stenting was performed to relieve the obstruction, as depicted in Figure 1. Postprocedure, she had brisk urine output, with daily output ranging from 2.5 to 3.5 L. Adequate hydration was ensured to balance the post-obstructive diuresis. Creatinine started trending down besides improving the urine output. The patient improved completely and was discharged successfully. On further follow-up, the stent was removed with the return of creatinine to baseline, as seen in Figure 1.

## Discussion

Our case demonstrated that carboplatin induced urothelial injury, resulting in hematuria. Platinum coordination complexes have broad antineoplastic activity and become the foundation for the treatment of ovarian, head and neck, bladder, esophagus, lung, and colon cancers. Platinum compounds enter the cell via Cu2+ transporter CTR1. Inside the cell, water molecules displace the chloride, cyclohexane, or oxalate ligand of the analog, resulting in a positively charged molecule. This molecule then reacts with nucleophile sites on DNA and proteins. The molecular mechanisms attributed involve the formation of 1,2 intrastrand crosslinks of purine bases, resulting in the subsequent blockade of cell division. Additionally, it leads to the generation of reactive oxygen species, which induce lipid peroxidation, and ultimately triggers cell apoptosis [5]. The DNA-platinum adducts inhibit DNA replication and transcription, which leads to the formation of single- and double-stranded breaks, as well as miscoding. If these adducts are recognized by p53 and other checkpoint proteins, they induce apoptosis. Aquation of cisplatin is favored at low concentrations of chloride inside the cell and in the urine. High concentrations of chloride stabilize the drug, explaining the effectiveness of chloride diuresis in preventing nephrotoxicity. Adduct formation is an important predictor of response.

Basal-type breast cancers like BRCA1 and BRCA2 mutation and triple-negative cancers are uniquely susceptible to platinum compounds [6]. Commonly, carboplatin is administered intravenously over 25 minutes. Carboplatin has less protein bounding than cisplatin with major drug elimination in the kidney (t1/2-2 hours). Carboplatin is a choice when severe nausea and impaired renal function limit the use of cisplatin. In our case, hematuria occurred after the initiation of carboplatin. Carboplatin is indeed known for being less nephrotoxic than cisplatin, which means it is less likely to cause kidney damage. However, carboplatin can still lead to certain kidney-related side effects, such as hematuria, although this is not a common side effect. The dose adjustment of carboplatin is done with the Calvert formula: dose (mg) = Target AUC x (GFR +25) [7]. Myelosuppression, particularly thrombocytopenia, is the dose-limiting toxicity of carboplatin.

Based on the Calvert formula (with an area under the curve [AUC] of 6), our patient received 700 mg of carboplatin. Ettinger et al. used carboplatin (336 mg/m^2^/day) in pediatric AML patients [8]. They observed a patient with gross hematuria who had previously received cyclophosphamide without any complications. The condition was managed conservatively. Agraharkar et al. reported a similar case in an ovarian malignancy where carboplatin-induced hematuria resulted in obstructive AKI [9]. The patient received 1,100 mg carboplatin (753 mg/m^2^) and 250 mg paclitaxel (175 mg/m^2^). The patient was managed conservatively, and the obstruction resolved spontaneously with an improvement in renal function. In our case, there was no concurrent use of cyclophosphamide, and the mechanism that would have contributed to hematuria is the sloughing of uroepithelium. Taj et al. encountered a similar case of a 34-year-old female with basal type of breast carcinoma who had gross hematuria after two days of completion of carboplatin chemotherapy. The patient had normal creatinine, and the patient was managed conservatively [10]. Although carboplatin can cause thrombocytopenia, our patient had normal platelets. Aggressive hydration, as typically done with cisplatin, was not carried out in this case.

The management of carboplatin-induced hematuria with obstructive AKI primarily involves supportive measures to maintain renal function and alleviate obstructive symptoms. These may include hydration, diuretics, and the use of medications to control pain and inflammation. In severe cases where urinary obstruction is significant, interventions such as ureteral stenting or percutaneous nephrostomy may be necessary to restore urine flow and relieve symptoms. To prevent carboplatin-induced hematuria, patients should be closely monitored during carboplatin therapy. Adequate hydration and urinary alkalinization may help reduce the risk of urothelial toxicity. Dose adjustments or alternative therapies may be considered for patients at higher risk of developing hematuria. Newer drugs like dicycloplatin are more promising and have fewer side effects as compared to cisplatin and carboplatin. Since it is a single case report, further studies need to be evolved to have a better understanding of the effect of the drug.

## Conclusions

Although cisplatin is associated with more nephrotoxicity, carboplatin can also cause renal adverse effects. Carboplatin induces cytotoxicity leading to hematuria and clots in the ureter with obstructive AKI. Adequate hydration before infusing carboplatin can help mitigate renal complications. In conclusion, carboplatin-induced hematuria with obstructive AKI is a rare but serious complication that can occur during chemotherapy. We should be vigilant in monitoring patients receiving carboplatin therapy for signs of this complication and be prepared to intervene promptly to prevent further kidney damage and improve patient outcomes.
